# QT Prolongation and Torsades De Pointes Due to Undiagnosed Sheehan Syndrome: A Rare Cause of Lethal Arrhythmia

**DOI:** 10.7759/cureus.85974

**Published:** 2025-06-13

**Authors:** Yuto Oshizaka, Reina Suzuki, Shunsuke Funasaki, Junji Shiotsuka, Hidenori Sanayama

**Affiliations:** 1 Department of Comprehensive Medicine 2, Division of Anesthesiology and Critical Care Medicine, Saitama Medical Center, Jichi Medical University, Saitama, JPN; 2 Department of Comprehensive Medicine 1, Division of General Medicine, Saitama Medical Center, Jichi Medical University, Saitama, JPN; 3 Department of Comprehensive Medicine 1, Division of Endocrinology and Metabolism, Saitama Medical Center, Jichi Medical University, Saitama, JPN; 4 Department of Anesthesiology and Critical Care Medicine, Jichi Medical University, Tochigi, JPN

**Keywords:** adrenal crisis, hypopituitarism, hypothyroidism, qt prolongation, sheehan syndrome

## Abstract

We report a case of long QT syndrome (LQTS) associated with torsades de pointes (TdP) in a 57-year-old female patient transferred to our cardiac care unit/intensive care unit with septic shock secondary to urinary tract infection (UTI) from an adjacent hospital. The patient had no notable medications or electrolyte abnormalities that could have contributed to acquired LQTS, with normal potassium and only slightly reduced calcium (1.06 mmol/L) and magnesium (1.8 mg/dL). After detailed history taking and exploration, the patient was diagnosed with Sheehan syndrome secondary to a complicated delivery she experienced 20 years before. The diagnosis was confirmed by a combination of clinical history (poor lactation after delivery, progressive lethargy), laboratory findings showing anterior pituitary hormone deficiencies, and imaging evidence of empty sella on pituitary MRI. The diagnosis had been missed for two decades due to a lack of regular medical checkups and the gradual onset of symptoms that went unrecognized. Hypothyroidism and adrenal crisis precipitated by sepsis were considered as the underlying etiology for her LQTS-associated TdP. Concurrent supplementation with levothyroxine and hydrocortisone for two weeks successfully shortened the QTc interval from 722 msec to 434 msec, with sustained normalization thereafter.

## Introduction

Sheehan syndrome, first described by Harold L. Sheehan in 1937 [1], is a form of hypopituitarism caused by ischemic necrosis of the anterior pituitary gland following severe postpartum hemorrhage. This condition typically develops when massive blood loss during or after delivery leads to hypovolemic shock, resulting in insufficient blood supply to the enlarged pituitary gland during pregnancy. Although the frequency of Sheehan syndrome has declined in developed countries due to modern obstetric care, it remains a significant health issue globally, with a prevalence ranging from 2.6 to 5.1 per 100,000 women in developed countries to much higher rates in developing regions [1]. Clinical manifestations of Sheehan syndrome can be variable and may include failure of lactation, secondary amenorrhea, fatigue, and signs of multiple hormone deficiencies. The diagnosis is often delayed for years due to the gradual onset of symptoms and their nonspecific nature. The syndrome is often reported to present with ECG abnormalities such as T-wave inversion, ST depression, and prolonged QT intervals [2-4]. These abnormalities may be attributed to various mechanisms, including electrolyte imbalances (particularly hypokalemia and hypomagnesemia), direct effects of hormone deficiencies on myocardial repolarization, or catecholamine surges secondary to hypoglycemia [3,4]. Both hypothyroidism and adrenal insufficiency can prolong the QT interval through hormonal effects on cardiac ion channels and repolarization processes, potentially leading to life-threatening arrhythmias [5-8]. While most cardiac manifestations are mild and reversible, life-threatening arrhythmias such as ventricular fibrillation (VF) and torsades de pointes (TdP) have been reported in rare cases, presenting significant diagnostic and management challenges. TdP as a manifestation of long-standing undiagnosed Sheehan syndrome is exceptionally rare and may be easily overlooked without a high index of clinical suspicion.

The management of Sheehan syndrome with cardiac arrhythmias presents a unique clinical dilemma [3]. The conventional approach to treating TdP includes the correction of electrolyte abnormalities and the administration of anti-arrhythmic medications. However, in Sheehan syndrome, these arrhythmias can occur in the absence of significant electrolyte disturbances and may be primarily driven by hormone deficiencies [3]. This raises important questions regarding optimal treatment strategies, including whether hormone replacement therapy should be prioritized over standard anti-arrhythmic approaches, and regarding the optimal timing and duration of hormone supplementation. Currently, there is no clear consensus on the optimal timing, sequence, and duration of hormone supplementation in such cases [3,9].

Herein, we present a case of a 57-year-old woman who presented with long QT syndrome (LQTS)-associated TdP due to undiagnosed Sheehan syndrome for 20 years after delivery but without significant electrolyte abnormalities, whose QT interval shortened successfully after administration of glucocorticoid and levothyroxine. This case highlights the importance of considering endocrine disorders in the differential diagnosis of unexplained arrhythmias, especially in female patients with relevant obstetric history, and demonstrates the dramatic resolution of QT interval prolongation with appropriate hormone replacement therapy.

## Case presentation

A 57-year-old Japanese woman was brought to a nearby hospital by ambulance due to a one-day history of a fever of 39°C followed by the development of impaired consciousness. She had never had a medical checkup for more than 10 years, but her husband noted she had two ER visits due to coma secondary to severe hypoglycemia within the previous 12 months. She did not take any medications, supplements, herbals, or over-the-counter drugs at home. She did not smoke, drink, or use illicit drugs. She was a housewife, had one child, and lived with her husband and child. Her family history was unremarkable. On examination, she was lethargic and hypotensive. The laboratory tests showed a low blood glucose level of 20 mg/dL, serum sodium of 135 mEq/L, potassium of 3.5 mEq/L, calcium of 1.09 mmol/L, and pyuria. Magnesium levels were not measured at the referring hospital. She was treated with piperacillin/tazobactam 2.25 g q8hr for septic shock due to a urinary tract infection (UTI). Blood cultures remained negative, but urine cultures grew *Escherichia coli*. On the following day, she developed episodes of arrhythmia with transitions between ventricular tachycardia (VT) and VF, which lasted for 30 seconds or more at the longest. The referring hospital documented these episodes as VT and VF without specifically identifying TdP as the underlying mechanism. Defibrillation was not administered as the patient promptly responded to resuscitative measures and regained adequate circulation. Following the onset of VT and VF, lidocaine and bisoprolol tape 4 mg were administered as antiarrhythmic treatment, but the episodes persisted. She also developed rhabdomyolysis and acute renal failure. She was transferred to the cardiac care unit (CCU) of our tertiary care hospital for further evaluation and care.

On arrival at our CCU, the patient looked extremely lethargic, and the history was limited due to the patient's impaired consciousness. The examination revealed a temperature of 36.8°C, blood pressure of 84/48 mmHg, pulse rate of 86 bpm regular, and respiratory rate of 26 breaths per minute with SpO_2_ 100% on room air. Her body weight was 45 kg, and her body mass index was 19.2 kg/m^2^. The Glasgow Coma Scale was E4V5M6, though she was not able to make eye contact with the team. Upon further physical examination, the absence of axillary hair was noted, while pubic hair was preserved (Note: upon follow-up interview after her recovery, the patient herself confirmed that she had lost her axillary hair after childbirth 20 years ago). The remainder of the physical examination was unremarkable. An abdominal contrast-enhanced CT performed to identify the source of infection revealed atrophic adrenal glands, with the left adrenal measuring only 5 mm in maximum thickness and the right adrenal gland being unidentifiable.

**Figure 1:**
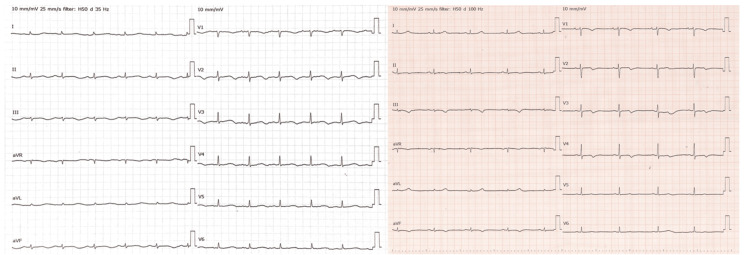
Electrocardiogram findings Left panel: 12-lead ECG on arrival with a prolonged QTc of 722 msec. Right panel: 12-lead ECG on hospital day 14 with a shortened QTc of 434 msec.

Her 12-lead ECG showed a prolonged, corrected QT interval of QTc 722 msec and T-wave inversion in the leads V2-V6, supporting TdP. Low voltage was observed in both limb and precordial leads, but poor R wave progression was not present (left panel, Figure 1). Detailed rhythm analysis revealed sinus rhythm with occasional premature ventricular contractions (PVCs) falling on the T wave (R-on-T phenomenon), which is a known trigger for TdP. No distinct U waves were identified. The results of the laboratory tests on hospital day 1 are summarized in Table 1. An echocardiography performed by our cardiologist was unremarkable with no wall motion abnormalities. While continuing bisoprolol tape 4 mg, intravenous magnesium sulfate administration was initiated for mild hypomagnesemia, and VT did not occur on the first day after transfer.

**Table 1:** The blood tests taken when the patient arrived at our CCU (hospital day 1) CK: creatine kinase; BUN: blood urea nitrogen; SCr: serum creatinine; Trop: troponin; CCU: cardiac care unit

Laboratory parameters	Values	Reference range
Na^+ ^(mEq/L)	136	138-146
K^+ ^(mEq/L)	3.7	3.6-4.9
Cl^-^ (mEq/L)	99	99-109
Mg^2+ ^(mg/dL)	1.7	1.8-2.4
Ca^2+ ^(mmol/L)	1.06	1.15-1.33
CK-MM (U/L)	8307	45-163
CK-MB (ng/mL)	20	<12
BUN (mg/dL)	35	8.0-22
SCr (mg/dL)	2.28	0.4-0.7
Trop-I (pg/mL)	227	<26.2

**Figure 2:**
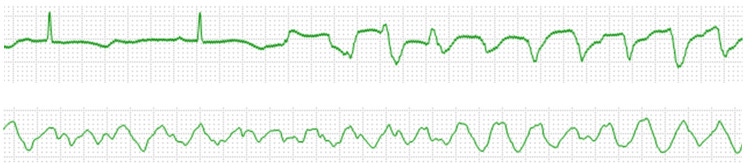
VT and VF episodes of monitor ECG Upper panel: VT episode considered related to TdP mechanism; lower panel: identified VF waveform. VT: ventricular tachycardia; VF: ventricular fibrillation; ECG: electrocardiogram; TdP: torsades de pointes

On hospital day 2, she developed frequent VT episodes considered related to the TdP mechanism and VF, with five VF episodes occurring within one hour (Figure 2). This represented a new cluster of arrhythmic events after a period of relative stability following her transfer to our facility, despite ongoing electrolyte repletion. This recurrence of life-threatening arrhythmias despite conventional management prompted more aggressive intervention. To avoid slower heart rates which may result in increasing heterogeneity of ventricular repolarization, more opportunities for PVCs and succeeding TdP, a temporal pacemaker (tPM) was implanted urgently and overdrive pacing was started at 100 beats/minute. The pacing rate of 100 bpm was determined as the minimum rate needed to suppress PVC episodes while maintaining stable hemodynamics. Amiodarone was not used given the risk of further prolonged QTc. Additionally, we discontinued bisoprolol due to concerns that beta-blockade might worsen the underlying pathophysiology.

As her clinical presentation was atypical for acquired LQTS, the team took a further clinical history from her husband. It was found that her activity and appetite had gradually decreased since a long vaginal delivery she experienced 20 years before. The husband did not recall a massive bleeding episode during the delivery. Although we attempted to obtain her medical records from that time, they were unavailable, so postpartum hemorrhage could not be definitively confirmed from documentation. Per the husband, the patient also had poor lactation, and she fed the baby with formula. The husband noted that she had become even more lethargic over the past five years. Upon follow-up interview after her recovery, the patient confirmed that she had continued to have regular menstrual cycles following delivery, without amenorrhea or significant irregularities, until she experienced natural menopause at age 51. In this context, Sheehan syndrome was suspected as the underlying cause of LQTS-associated TdP. The endocrine panel from blood samples collected on the early morning of hospital day 2 under fasting conditions revealed hypopituitarism (Table 2), which supported the diagnosis of Sheehan syndrome in conjunction with the additional history obtained from the husband.

**Table 2:** The endocrine panel on hospital day 2 (early morning) ACTH: adrenocorticotropic hormone; TSH: thyroid-stimulating hormone; FT3: free T3; FT4: free T4; PRL: prolactin; LH: luteinizing hormone; FSH: follicle-stimulating hormone; GH: growth hormone

Laboratory parameters	Values	Reference range
ACTH (pg/mL)	<1.5	7.2-63.3
Cortisol (µg/dL)	0.77	6.24-18.0
TSH (mIU/L)	3.704	0.35-4.94
FT3 (pg/mL)	0.81	1.71-3.71
FT4 (ng/dL)	0.21	0.7-1.48
PRL (ng/mL)	0.39	4.9-29.3
LH (mIU/mL)	5.60	0.79-5.72
FSH (mIU/mL)	10.39	2.0-8.3
GH (ng/mL)	0.96	0.010-3.607

**Figure 3:**
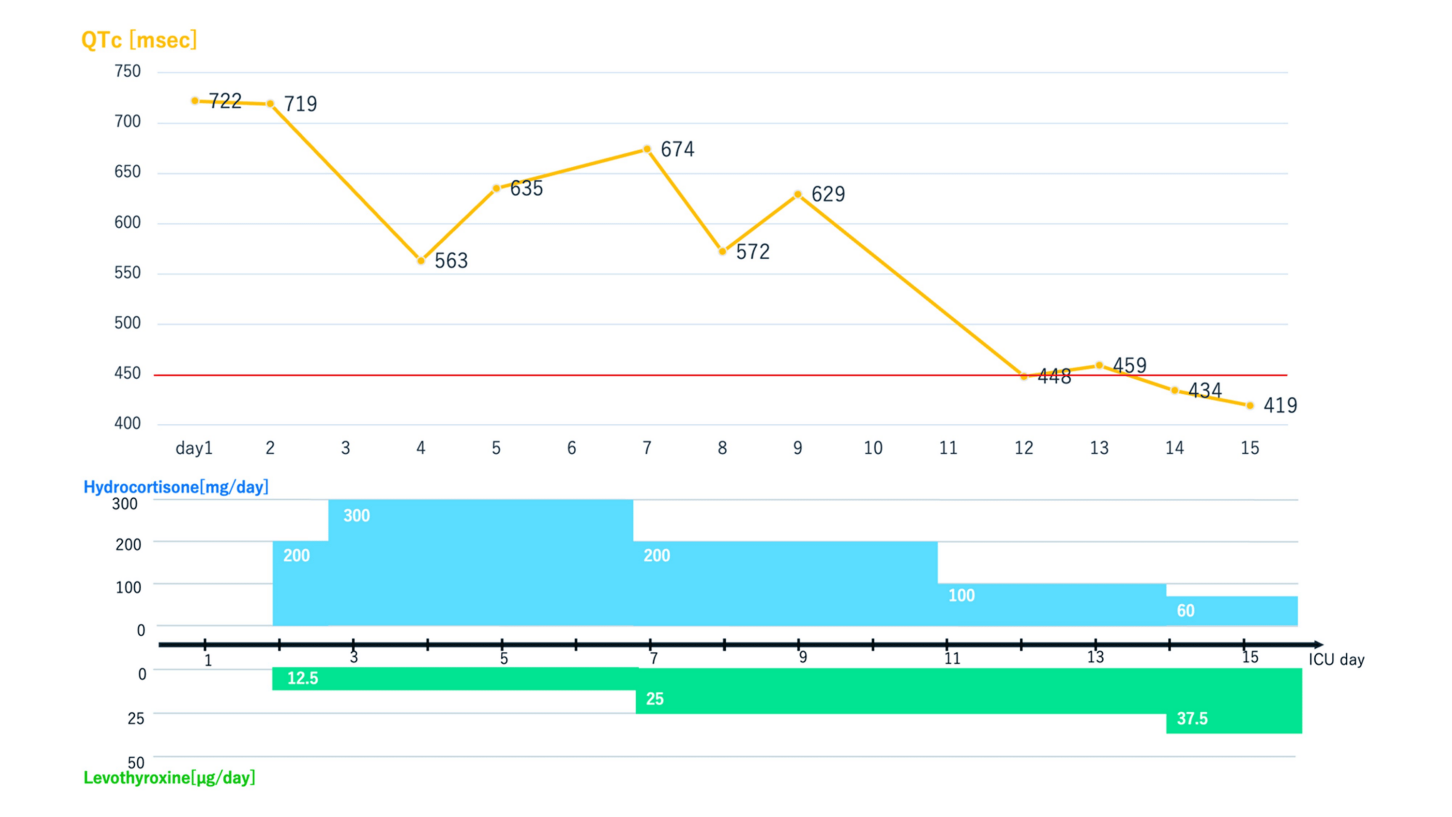
Summary of sequential QTc and hormones dosage The pacemaker was removed on day 14 after confirmation of no torsades de pointes under 24-hour backup pacing. Levothyroxine was increased biweekly while monitoring for complications of ischemic heart disease by echocardiography and 12-lead ECG; hydrocortisone was subsequently tapered off. As her general condition stabilized, she was transferred from the ICU on day 28.

For hypopituitarism, hydrocortisone and levothyroxine were initiated in consultation with the endocrinology department. To minimize the risks of adrenal crisis, we administered levothyroxine after hydrocortisone and also with the lowest dose. The doses of hydrocortisone and levothyroxine were summarized with daily QTc in Figure 3. Hydrocortisone was increased to account for sustained hypotension and then tapered cautiously by monitoring signs of hypoperfusion such as blood pressure, SvO_2_, urine output, and serum lactate levels. The levothyroxine dose was increased by 12.5 µg/day per week cautiously by monitoring thyroid hormone levels and cardiac complications. Thyroid function and adrenal function were monitored weekly, while cardiac biomarkers, including aspartate transaminase (AST), creatine phosphokinase (CPK), and creatine kinase (CK)-MB, were assessed daily during the ICU stay to guide treatment decisions. Troponin-I was monitored until hospital day 2, when a declining trend was confirmed, after which routine monitoring was discontinued.

After hospital day 5, we paused the temporary pacemaker for at least an hour every day to check the native cardiac rhythm and QTc on a 12-lead ECG. Pacing stability was monitored closely, and on hospital day 5, it recurred when the pacing position shifted and became unstable, confirming the continued need for pacing support. On hospital day 8, the patient resumed sinus rhythm. On hospital day 12, QTc was shortened to 448 msec and backup pacing was turned off. After confirming the absence of TdP under a 24-hour backup pacing, the tPM was removed on hospital day 14 (day 13 of hormone replacement).

**Figure 4:**
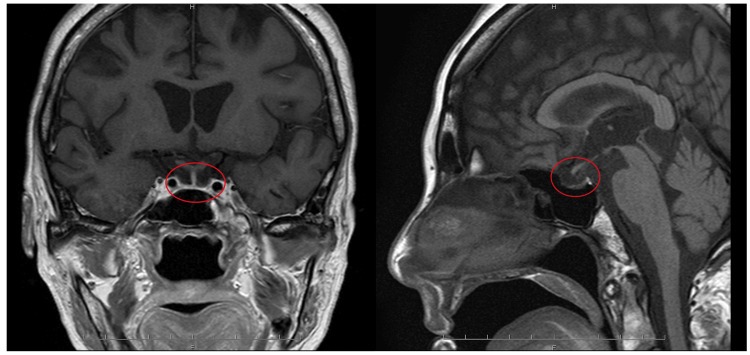
Pituitary MRI taken on day 28 (T1WI) Left panel: Coronal gadolinium-enhanced T1WI. Right panel: Sagittal non-contrast T1WI. The structures within the red circles represent the pituitary gland. Both images demonstrate a flattened pituitary gland compressed against the floor of the sella turcica, consistent with an empty sella. The posterior pituitary bright spot is preserved, and there is no deviation of the pituitary stalk. T1WI: T1-weighted imaging

A gadolinium-enhanced pituitary MRI in Figure 4, obtained on hospital day 28 upon improvement in her general conditions, showed empty sella, which supported the diagnosis of Sheehan syndrome. The findings were consistent with partial hypopituitarism rather than complete pituitary destruction, as evidenced by the preserved posterior pituitary bright spot and normal pituitary stalk position. Other causes of empty sella, such as pituitary tumors or previous surgery, were excluded based on the clinical history. On the same day, the patient was transferred to the floor. Hydrocortisone and levothyroxine were then tapered carefully by the endocrinology department.

On hospital day 83, the patient was transferred to a rehabilitation hospital for further rehabilitation on hydrocortisone 10 mg/day and levothyroxine 37.5 µg/day. At a five-month follow-up, the patient had achieved complete neurological recovery with independent living capacity, allowing for discharge to home. A comprehensive endocrine stimulation test was performed six months later after discontinuing cortisol and levothyroxine for 24 hours. Corticotropin-releasing hormone (CRH), thyrotropin-releasing hormone (TRH), and luteinizing hormone-releasing hormone (LH-RH) stimulation tests were conducted (Table 3). The CRH stimulation test showed no response in both adrenocorticotropic hormone (ACTH) (<1.5 pg/mL throughout) and cortisol (<0.2 µg/dL throughout). A rapid ACTH stimulation test was subsequently performed 24 hours later, which also showed no cortisol response (<0.2 µg/dL throughout), confirming complete hypothalamic-pituitary-adrenal (HPA) axis suppression. In contrast, thyroid-stimulating hormone (TSH) responded appropriately to TRH stimulation (from 3.612 to 9.965 mIU/L at 30 minutes), indicating preserved hypothalamic-pituitary-thyroid axis function. Luteinizing hormone (LH) and follicle-stimulating hormone (FSH) showed normal responses to LH-RH stimulation. Insulin-like growth factor I (IGF-1) measured seven months later was 47 ng/mL (age-adjusted reference range: 72-205 ng/mL). While growth hormone levels were within the normal range at presentation, the reduced IGF-1 may reflect either age-related physiological decline or subtle growth hormone axis dysfunction in the context of panhypopituitarism.

**Table 3:** Combined TRH, CRH, and LH-RH stimulation test results at six-month follow-up ACTH: adrenocorticotropic hormone; TSH: thyroid-stimulating hormone; PRL: prolactin; LH: luteinizing hormone; FSH: follicle-stimulating hormone; TRH: thyrotropin-releasing hormone; CRH: corticotropin-releasing hormone; LH-RH: luteinizing hormone-releasing hormone

Laboratory parameters	Values			
	Pre	Post 30 minutes	Post 60 minutes	Post 90 minutes
ACTH (pg/mL)	<1.5	<1.5	<1.5	-
Cortisol (µg/dL)	<0.2	<0.2	<0.2	<0.2
TSH (mIU/L)	3.612	9.965	8.312	-
PRL (ng/mL)	0.39	0.92	0.70	-
LH (mIU /mL)	5.44	16.88	17.22	18.01
FSH (mIU /mL)	15.42	-	20.96	22.95

The electrocardiogram recorded at the same time showed a QTc of 410 msec and a disappearance of low QRS voltage, demonstrating sustained normalization of cardiac repolarization with hormone replacement therapy. Now, the patient is maintained on hydrocortisone 10 mg/day and levothyroxine 37.5 µg/day without major adverse effects.

## Discussion

Two important clinical issues arose from this patient, who presented with LQTS-associated TdP with underlying, undiagnosed Sheehan syndrome. First, Sheehan syndrome could present with lethal ventricular arrhythmia without notable electrolyte disturbances. Second, the QT interval could normalize with hormone replacement (corticosteroid and thyroid hormones in the present patient), but it could take time in the order of weeks. This time lag suggests the importance of continuing the treatment while awaiting the treatment effect.

Diagnostic approach and reasoning

Our diagnostic approach followed a systematic process: first, we identified unexplained LQTS-associated TdP without significant electrolyte abnormalities or medication-induced causes. Second, we obtained a detailed history from her husband revealing poor lactation after delivery and progressive lethargy spanning two decades, which raised suspicion of Sheehan syndrome. Third, our endocrine testing confirmed panhypopituitarism with low levels of ACTH, cortisol, TSH, and free T4, consistent with our clinical suspicion. Fourth, pituitary MRI demonstrated empty sella, further supporting the diagnosis. Additionally, abdominal CT revealed atrophic adrenal glands consistent with chronic adrenal insufficiency. Anti-thyroid peroxidase (anti-TPO) and anti-thyroglobulin antibodies measured after discharge were negative, helping distinguish between pituitary and primary autoimmune causes of thyroid dysfunction. While empty sella can be seen in various conditions, the combination of obstetric history, clinical course, laboratory evidence of panhypopituitarism, negative thyroid autoantibodies, and positive response to hormone replacement therapy conclusively established Sheehan syndrome as the definitive diagnosis.

Sheehan syndrome and lethal arrhythmia

While reports on ECG changes with hypothyroidism are widely available, such information is limited for hypopituitarism. The largest report, which studied 20 patients with hypopituitarism and included three patients with Sheehan syndrome in the 1960s, found that the most common ECG changes were inversion or flattening of T waves, low voltage of QRS complexes, ST segment depression, and QT prolongation in the order of the frequency [2]. However, this limited historical data underscores the need for more contemporary case series and mechanistic studies. Not many reports delved into the etiologies of these ECG changes in hypopituitarism; however, electrolyte disturbances, catecholamine surge secondary to hypoglycemia, and hormone deficiencies (hypothyroidism, ACTH) were considered possible causes [3,4]. More recently, hypokalemia (62.5%), hypoglycemia (37.5%), and hypomagnesemia (25%) were reported to be the most common laboratory abnormalities among the eight established cases with Sheehan syndrome presenting with VT [3].

In the present patient, as there were no notable electrolyte abnormalities that would have caused LQTS (although serum magnesium was slightly low, QT prolongation persisted for two weeks after magnesium correction) and QTc shortened markedly after hormone replacement, it was hypothesized that QT prolongation was driven by a deficiency of either adrenal cortical hormone or thyroid hormone or both. While specific mechanisms of association between LQTS and adrenal/thyroid hormones are yet to be determined and may be multifactorial, possible hypotheses for LQTS in the present patient could be made as follows.

Glucocorticoids and QT Interval

Glucocorticoids regulate the expression of serum- and glucocorticoid-inducible kinase-1 (SGK1) genes. SGK1 up-regulates potassium voltage-gated channel subfamily E member 1/potassium voltage-gated channel subfamily Q member 1 (KCNE1/KCNQ1) and human ether-a-go-go related gene (hERG), resulting in an increase in outward potassium current, which promotes myocardial repolarization and shortens QT interval under physiological stress [5]. The cortisol deficiency in the present patient may have reduced SGK1 expression, resulting in a reduction in delayed outward potassium currents, which may have prolonged the depolarization time of cardiomyocytes and led to prolonged QTc. The fact that KCNQ1 is one of the causative genes for LQTS1, a congenital QT prolongation syndrome, may support this hypothesis [6].

Thyroid Hormones and TdP

Prolongation of the QT interval in patients with hypothyroidism reportedly occurs in 21-33%, but complications of ventricular arrhythmias such as TdP are rare [7]. It has been suggested that thyroid hormones control the expression of potassium channels such as Kv1.5 or Kv4.2. Furthermore, thyroid hormones may also play an important role in the inhibition of TdP by suppressing early afterdepolarizations (EAD). Thyroid hormones down-regulate Na^+^/Ca^2+^ exchanger 1 (NCX1), and it has been reported that in an intact rabbit heart model of LQTS, inhibition of NCX1 was effective in preventing TdP due to a suppression of EADs [8]. It could be hypothesized that NCX1 causes EADs due to the inward current generated when Ca^2+^ concentration is increased after a spontaneous release of Ca^2+^ from the sarcoplasmic reticulum.

These hypotheses are not mutually exclusive, and a synergistic effect of combined hormone deficiencies may be more plausible in Sheehan syndrome, as both glucocorticoids and thyroid hormones influence cardiac repolarization through complementary pathways [5-8]. Additionally, while serum magnesium was only mildly reduced and QT prolongation persisted despite repletion, intracellular magnesium dynamics may still contribute to arrhythmogenesis, as serum levels do not always reflect total-body magnesium status [3].

Considering these hormones are released in response to physiological stress, the risk of fatal arrhythmias in patients with hypopituitarism could increase under excessive stresses such as sepsis. Indeed, in a previous report, a patient with Sheehan syndrome who was suffering from influenza experienced a fatal arrhythmia [10]. In the present patient, a UTI leading to sepsis might have acted as a similar physiological stressor. Although sepsis induces endogenous catecholamine elevation as part of the stress response [11], which could potentially contribute to arrhythmogenesis, the persistent arrhythmias despite normalized metabolic parameters and the subsequent resolution with hormone replacement therapy suggest that hormone deficiencies rather than catecholamine surge played the more dominant role in our patient's TdP. These examples may encourage clinicians to add hypopituitarism-associated lethal arrhythmias to their differential diagnoses when they encounter patients with lethal arrhythmias without clear-cut causes.

Hormone replacement therapy and treatment duration for LQTS due to hypopituitarism

While there is no consensus for the treatment of lethal ventricular arrhythmias specifically due to hypopituitarism, hormonal replacement seems to be the therapeutic key, and other treatment options, e.g., electrolyte repletion and antiarrhythmic drugs, may be considered as adjuncts in these situations [2,3]. There are reports where electrolyte replacement failed to normalize QTc, like in the present patient [3], supporting the need for hormone replacement as the cornerstone of the treatment. In the present patient, the QT interval normalized after 12 days from the initiation of hydrocortisone and levothyroxine. The types of hormones administered in the previously reported cases of hypopituitarism complicated by lethal arrhythmias varied widely depending on the deficient hormones [2,3]. In cases of hypopituitarism with both ACTH and thyroid hormone deficiency in the literature, QT time was reported to shorten in 2-4 weeks with the replacement of both hormones [3,9], which is consistent with the clinical course of the present patient.

Clinicians, however, should note that treatment duration may vary depending on the type of hormone deficiency. Some reports describe that in LQTS associated with adrenal crisis, QTc was normalized within a few days to weeks after the initiation of glucocorticoid replacement [10]. On the other hand, it was reported that several months to a year were required for the ECGs to normalize in cases of LQTS associated with hypothyroidism without electrolyte abnormalities [7,12,13]. In the largest report that discussed ECG changes in 20 patients with hypopituitarism, glucocorticoid supplementation alone normalized QTc in some patients with hypopituitarism, while the other patients’ ECG changes were refractory to treatment until thyroid hormone replacement was started [2]. To minimize the risks of adrenal crisis, it would be safer to begin with corticosteroid replacement alone before giving thyroid hormone [14]. However, clinicians should be aware that this sequential approach may potentially delay full QTc normalization in patients requiring both hormones, requiring careful risk-benefit assessment. Additionally, conventional antiarrhythmic drugs may pose risks in hormonally driven LQTS by further prolonging QT intervals and are usually resistant to such therapy [15], emphasizing why hormone replacement should be prioritized over standard antiarrhythmic approaches in documented hormone deficiency-induced arrhythmias. It should be noted, however, that administration of thyroid hormones may be required in certain patients with LQTS, specifically where the clinical/laboratory signs of hypothyroidism are evident.

It is important to acknowledge that our observations regarding treatment duration are based on a single case, and broader data on the precise timeline of QTc normalization in hypopituitarism-induced LQTS is lacking in the current literature. Individual patient factors, the severity of hormone deficiencies, concurrent medical conditions, and specific hormone replacement protocols could significantly influence treatment response time. Given the rarity of this condition, the accumulation of similar case reports and clinical experiences will likely be the primary source of evidence to guide future management and establish expected treatment timelines in this specific clinical scenario.

Delayed presentation of Sheehan syndrome: the importance of maintaining clinical suspicion decades after delivery

An interesting aspect of this case worth highlighting is the patient's survival for approximately 20 years without hormone replacement despite underlying Sheehan syndrome. This prolonged period without treatment is particularly notable given the atypical presentation - our patient did not experience massive postpartum hemorrhage, which is classically associated with Sheehan syndrome. As Diri et al. [1] noted in their comprehensive review, while massive postpartum hemorrhage is the typical etiology, rare cases of hypopituitarism without severe postpartum hemorrhage have been reported. Similarly, Taniguchi et al. [16] reported a case of Sheehan syndrome remaining undiagnosed for 18 years after postpartum hemorrhage, with the patient surviving until influenza A infection precipitated the adrenal crisis. This case further illustrates the potential for prolonged survival with partial hypopituitarism and emphasizes how acute physiological stress can unmask previously compensated endocrine dysfunction. The sequential nature of pituitary hormone loss - where gonadotropins are typically affected first and ACTH is preserved the longest - likely enabled our patient to maintain minimal but sufficient cortisol production until the stress of sepsis overwhelmed this tenuous endocrine balance. Our patient's clinical history shows a gradual progression: she experienced poor lactation after delivery (suggesting prolactin deficiency), gradually declining activity, and increasing fatigue over two decades (suggesting progressive thyroid and/or adrenal insufficiency), while menstruation continued for some time after delivery (indicating initially preserved gonadotropin function).

This case reminds clinicians that Sheehan syndrome should remain in the differential diagnosis for patients with unexplained cardiac arrhythmias even without a history of massive postpartum hemorrhage, as pituitary dysfunction can develop through other mechanisms such as small vessel ischemia or autoimmune processes, and patients with partial hypopituitarism may survive for extended periods without hormone replacement before experiencing acute decompensation during periods of physiological stress. While this case focuses on Sheehan syndrome, the principles apply more broadly to other forms of hypopituitarism. This experience suggests that clinicians should consider endocrine disorders in the differential diagnosis of unexplained arrhythmias, particularly when conventional treatments are ineffective. Early endocrine workup in patients with unexplained QT prolongation and resistance to standard therapy could lead to more timely diagnosis and appropriate hormone replacement, potentially avoiding life-threatening arrhythmic complications.

## Conclusions

Endocrine disorders involving glucocorticoid and thyroid hormone deficiencies can significantly contribute to QT prolongation and potentially life-threatening arrhythmias. Our case demonstrates that hormone replacement therapy should be the primary approach in documented hormone deficiency-induced QT prolongation. Comprehensive endocrine evaluation should be included when investigating unexplained LQTS, particularly in women with relevant obstetric history. While our single case observation suggests QT interval normalization within approximately two weeks with appropriate hormone supplementation, treatment response likely varies between individuals, and further studies are needed to establish typical treatment timelines. These principles apply broadly to other forms of hypopituitarism when managing unexplained arrhythmias resistant to standard therapy.
